# Third generation cephalosporin resistance in clinical non-typhoidal *Salmonella enterica* in Germany and emergence of *bla*_CTX-M_-harbouring pESI plasmids

**DOI:** 10.1099/mgen.0.000698

**Published:** 2021-10-25

**Authors:** Michael Pietsch, Sandra Simon, Anika Meinen, Eva Trost, Sangeeta Banerji, Yvonne Pfeifer, Antje Flieger

**Affiliations:** ^1^​ Unit for Enteropathogenic Bacteria and Legionella and National Reference Centre for Salmonella and other Bacterial Enterics, Robert Koch Institute, Wernigerode, Germany; ^2^​ Unit for Gastrointestinal Infections, Zoonoses and Tropical Infections, Robert Koch Institute, Berlin, Germany; ^3^​ Unit for Nosocomial Pathogens and Antibiotic Resistances, Robert Koch Institute, Wernigerode, Germany

**Keywords:** *Salmonella enterica*, antibiotic resistance, extended-spectrum β-lactamase (ESBL), plasmid, pESI

## Abstract

Non-typhoidal *Salmonella enterica* is an important gastrointestinal pathogen causing a considerable burden of disease. Resistance to third generation cephalosporins poses a serious threat for treatment of severe infections. In this study occurrence, phylogenetic relationship, and mechanisms of third generation cephalosporin resistance were investigated for clinical non-typhoidal *S. enterica* isolates in Germany. From 2017 to 2019, we detected 168 unique clinical *S. enterica* isolates with phenotypic resistance to third generation cephalosporins in a nation-wide surveillance. Compared to previous years, we observed a significant (*P*=0.0002) and consistent increase in resistant isolates from 0.41 % in 2005 to 1.71 % in 2019. In total, 34 different serovars were identified, most often *S*. Infantis (*n*=41; 24.4 %), *S*. Typhimurium (*n*=27; 16.1 %), *S*. Kentucky (*n*=21; 12.5 %), and *S*. Derby (*n*=17; 10.1 %). Whole genome analyses revealed extended-spectrum β-lactamase (ESBL) genes as main cause for third generation cephalosporin resistance, and most prevalent were *bla*_CTX-M-1_ (*n*=55), *bla*_CTX-M-14_ (*n*=25), and *bla*_CTX-M-65_ (*n*=23). There was no strict correlation between serovar, phylogenetic lineage, and ESBL type but some serovar/ESBL gene combinations were detected frequently, such as *bla*_CTX-M-1_ and *bla*_CTX-M-65_ in *S*. Infantis or *bla*_CTX-M-14b_ in *S*. Kentucky. The ESBL genes were mainly located on plasmids, including IncI, IncA/C variants, emerging pESI variants, and a novel *bla*_CTX-M-1_harbouring plasmid. We conclude that third generation cephalosporin resistance is on the rise among clinical *S. enterica* isolates in Germany, and occurrence in various *S. enterica* serovars is most probably due to multiple acquisition events of plasmids.

## Data Summary

The authors confirm that all supporting data, code and protocols have been provided within the article. Illumina raw sequence reads were submitted to the European Nucleotide Archive (ENA) (https://www.ebi.ac.uk/ena/) and can be accessed under the bioproject PRJEB41165.

**Impact Statement**

Non-typhoidal *Salmonella enterica* (NTS) is one of the most important agents of foodborne infections worldwide. Although in most cases salmonellosis is self-limiting, antibiotic treatment may be required. However, antibiotic resistance in NTS has been a globally emerging problem, limiting treatment options of NTS infections and further presenting a considerable threat to the public health and food safety. Knowledge about the distribution of antibiotic resistance patterns and the genetic factors in *S. enterica* is therefore essential for appropriate treatment of these infections and for early intervention to prevent the spread of antibiotic resistance, starting at the level of food production. In a nation-wide surveillance of third generation cephalosporin resistant *S. enterica* in Germany, we found that third generation cephalosporin resistance is on the rise among clinical NTS isolates. Its occurrence in various *S. enterica* serovars is likely due to multiple acquisition events of plasmids highlighting its facilitated spread.

## Introduction

Non-typhoidal *Salmonella (S.) enterica* (NTS) is an important agent of foodborne infections [1]. In Europe and Germany, salmonellosis, after campylobacteriosis, represents the second most reported gastrointestinal infection caused by bacteria, encompassing 87 923 cases and 13 693 cases, respectively, in 2019 [2, 3]. *S*. Typhimurium and *S*. Enteritidis are the predominant serovars causing human infections in Germany and account for ~60 % of all reported cases (https://survstat.rki.de/). Further prevalent serovars like *S*. Infantis and *S*. Derby represented <5 % of the reported cases [3]. Although in most cases salmonellosis is self-limiting, antibiotic treatment may be required for risk group patients and more severe disease, like bloodstream infections [4]. Antibiotics for treatment of salmonellosis comprise β-lactams, fluoroquinolones, macrolides, and trimethroprim-sulfamethoxazole [4]. Challengingly, rising numbers of multidrug-resistant (MDR - resistant to three or more antimicrobials) *S. enterica* have been observed in recent years [5]. Including a slight increase in resistance to third generation cephalosporins, which has been reported in antimicrobial resistance surveillance studies for clinical isolates, and isolates from food (including retail meat) and animals [5–9].

Resistance to third generation cephalosporins in *S. enterica* is mainly due to the production of extended-spectrum β-lactamases (ESBLs) or AmpC β-lactamases [9, 10], with CTX-M-1 as the most prevalent ESBL type identified in previous studies [5, 11]. ESBL and AmpC genes are often located on plasmids, which facilitate their spread via horizontal gene transfer (HGT) between enterobacterial species and genera [12, 13]. Interestingly, ESBL production correlates with certain serovars in *S. enterica *. Among serovars found in human infections, ESBL genes have been more frequently detected in *S*. Typhimurium, *S*. Infantis and *S*. Kentucky than in *S*. Enteritidis [5, 14]. Furthermore, recent analyses on ESBL-producing *S*. Infantis from broiler chickens, broiler meat, and humans revealed a clonal lineage harbouring an especially successful conjugative pESI-like plasmid [15]. This raises the concern for transmission of ESBL genes in *S. enterica* along the food chain from the primary production systems to clinical cases.

In the present study, we assessed the resistance of clinical NTS in Germany and further analysed whole genomes of third generation cephalosporin resistant *S. enterica* for determination of phylogeny, resistance determinants, and genetic backbones to gain knowledge about the spread of this resistance mechanism in Germany.

## Methods

### Bacterial strains, serotyping, and antibiotic susceptibility testing

In the years 2017 – 2019, the National Reference Centre (NRC) for *Salmonella* and other Enteric bacterial Pathogens in Germany analysed 11 730 *S *. *enterica* isolates from human infections for pathogen surveillance. In the same time period 48 083 salmonellosis cases were notified to the German surveillance system. All *S. enterica* isolates were serotyped according to the White-Kauffmann-Le Minor scheme [16]. Further, antibiotic susceptibilities to 17 antibiotics (ampicillin, cefotaxime, ceftazidime, cefoxitin, meropenem, nalidixic acid, ciprofloxacin, gentamicin, amikacin, streptomycin, chloramphenicol, tetracycline and trimethoprim/sulfamethoxazole) were investigated by broth microdilution according to EUCAST criteria (v10.0, https://www.eucast.org/fileadmin/src/media/PDFs/EUCAST_files/Breakpoint_tables/v_10.0_Breakpoint_Tables.pdf). NTS isolates resistant to cefotaxime and/or ceftazidime according to EUCAST criteria (v10.0) were chosen for further analysis, however only one unique isolate per patient or infection cluster was considered. Among all NTS isolates from 2017 - 2019, 264 isolates showed phenotypic resistance to cefotaxime and/or ceftazidime and 166 unique isolates were selected for further analyses.

### Bacterial cultivation and whole genome sequencing

Müller Hinton broth was used for an overnight cultivation. DNA was subsequently extracted using the GenElute Bacterial Genomic DNA Kit (Sigma, St. Louis, Missouri, U.S.). DNA quantification was performed using the Qubit dsDNA HS Assay Kit (Thermo Fisher Scientific, Waltham, Massachusetts, U.S.) according to manufacturer’s instructions. Sequencing libraries were generated using the Nextera XT DNA Library Preparation Kit (Illumina, San Diego, CA, United States) and paired-end sequencing was performed using a MiSeq instrument with 2×300 MiSeq v3 reagent kit (Illumina, San Diego, CA, United States), HiSeq 2500 instrument with 2×150 with 2×300 HiSeq v2 RR reagent kit or NextSeq 550 instrument with 2×300 NextSeq v2.5 high reagent kit. Whole genome sequencing (WGS) was performed for 166 NTS isolates (Table S1, available with the online version of this article) and sequences were submitted to ENA (https://www.ebi.ac.uk/ena/) and can be accessed under the bioproject PRJEB41165.

### Bioinformatics analyses

Quality of the raw sequence data was assessed using FastQC v0.11.5 (https://github.com/s-andrews/FastQC). To verify taxonomic read classification, raw data was analysed with Kraken v0.10.6 [17]. The assembly pipeline of the analysis platform SeqSphere+ v6.0.7 (Ridom, Münster, Germany) was applied for *de novo* assembly using SPAdes v3.12.0 with default parameter [18], including an additional FastQC and read trimming step [19].

Multilocus sequence typing (MLST) and core genome MLST (cgMLST) were performed using the *de novo* assembled contigs and Ridom SeqSphere+ v6.0.7 (Ridom; Münster, Germany). The EnteroBase *S. enterica* cgMLST v2 scheme template was applied. *In silico* serotyping, based on the *de novo* assembly genomes, was conducted, using the serotype prediction tool SISTR [20].

*De novo* assembled contigs were used for resistance gene and plasmid replicon type identification. For the detection of acquired antimicrobial resistance genes, the tool Abricate in combination with the NCBIres Database was used (https://github.com/tseemann/abricate, [21]). *In silico* replicon typing was performed to assign resistance gene contigs to plasmid replicon sequences with Abricate using the PlasmidFinder v2.0 database [22]. Identified contigs with *bla* gene and plasmid replicon sequence were further investigated by means of Geneious Prime v2020.0.5 (Biomatters, Ltd., Auckland, New Zealand). In case of presence of a plasmid replicon sequence marker and a *bla* gene on an identical contig, these contigs were additionally checked for a possible chromosomal integration via *blastn* (database nucleotide collection (*nr/nt*)) and a subsequent Mauve alignment using *S*. Typhimurium str. LT2 as reference (NC_003197.2). If a chromosomal integration could be ruled out, *bla* gene carrying contigs were assigned to the respective identified plasmid replicon and considered as the *bla* resistance gene carrying contig. For phylogenetically closely related isolates with identical replicon and *bla* resistance gene patterns but exhibiting different *de novo* assembled contigs carrying *bla* gene and replicon sequence, a reconstruction strategy as described in Weber *et al.* was performed [23]. If multiple replicon sequences were identified in the genome sequence of an isolate and no replicon marker sequence could be addressed as genetic backbone for the *bla* gene or the *de novo* assembled *bla* gene carrying contigs exhibited not sufficient surrounding genetic environment, the *bla* gene carrying replicon was marked as ‘not determined’.

Plasmids carrying *bla*_CTX-M-1_ (*n*=22) and *bla*_CTX-M-65_ (*n*=15) in *S*. Infantis isolates were compared to variants of the emerging pESI plasmid [15]. In brief, contigs of these isolates were aligned and ordered according to the pESI plasmid backbone of the *S.* Infantis strain 119 944 (accession no. NZ_CP047882) using the MCM algorithm of Mauve [24]. These reordered plasmid drafts were subsequently compared to the pESI backbone and to a *bla*_CTX-M-65_ carrying pESI variant (pN17S0535, accession no. CP052810). Further *bla*_CTX-M-1_ harbouring plasmids of other serovars putatively belonging to the IncI1 replicon were compared to an IncI1 reference plasmid backbone (accession no. MK181566) using Mauve. Plasmids that carried AmpC gene *bla*_CMY-2_ were compared to IncA/C and IncI1 reference plasmids (accession no. CP012929, KT186369, CP015835, CP060509) using Mauve.

### Resistance transfer experiments and molecular typing

Transfer of third generation cephalosporin resistance was tested by broth mating for three selected isolates that harboured *bla*_CTX-M-3_ (18-04833), *bla*_CTX-M-65_ (19-02625) and *bla*_CTX-M-1_ (17-01817) using the sodium azide-resistant recipient strain *E. coli* J53 Azi^r^. Transconjugants were cultivated on LB agar with sodium azide (200 mg l^−1^) and ampicillin (30 mg l^−1^) and screened for resistance genes as previously described [11]. The plasmid sizes of *bla* gene positive transconjugants were determined by S1-nuclease restriction and pulsed-field gel electrophoresis [25]. Transconjugants and the isolates 18-00018 and 18-00680 were screened for resistance genes as previously described [11].

## Results

### Increasing proportion of third generation cephalosporin resistant *S. enterica* in Germany

In the years 2017 – 2019, the German National Reference Centre for *Salmonella* and other Enteric Bacterial Pathogens (NRC) analysed 11 730 NTS isolates from human infections (2017=3 399, 2018=3 706, 2019=4 625 isolates). Resistance to third generation cephalosporins was detected in 264 *S. enterica* isolates, of which most were collected in 2019 (2017: *n*=36, 2018: *n*=56, 2019: *n*=172). A substantial part of the isolates (*n*=100) from 2019 was attributed to a single infection cluster of *S*. Typhimurium in a regional setting. Four unique/representative isolates of this cluster which harboured certain changes (see details below) were included into the subsequent analyses. Consequently, the number of third generation cephalosporin resistant isolates considered for further analysis in 2019 was 76, resulting in a total of 168 study isolates for the years 2017–2019 (Table S1).

**Fig. 1.**
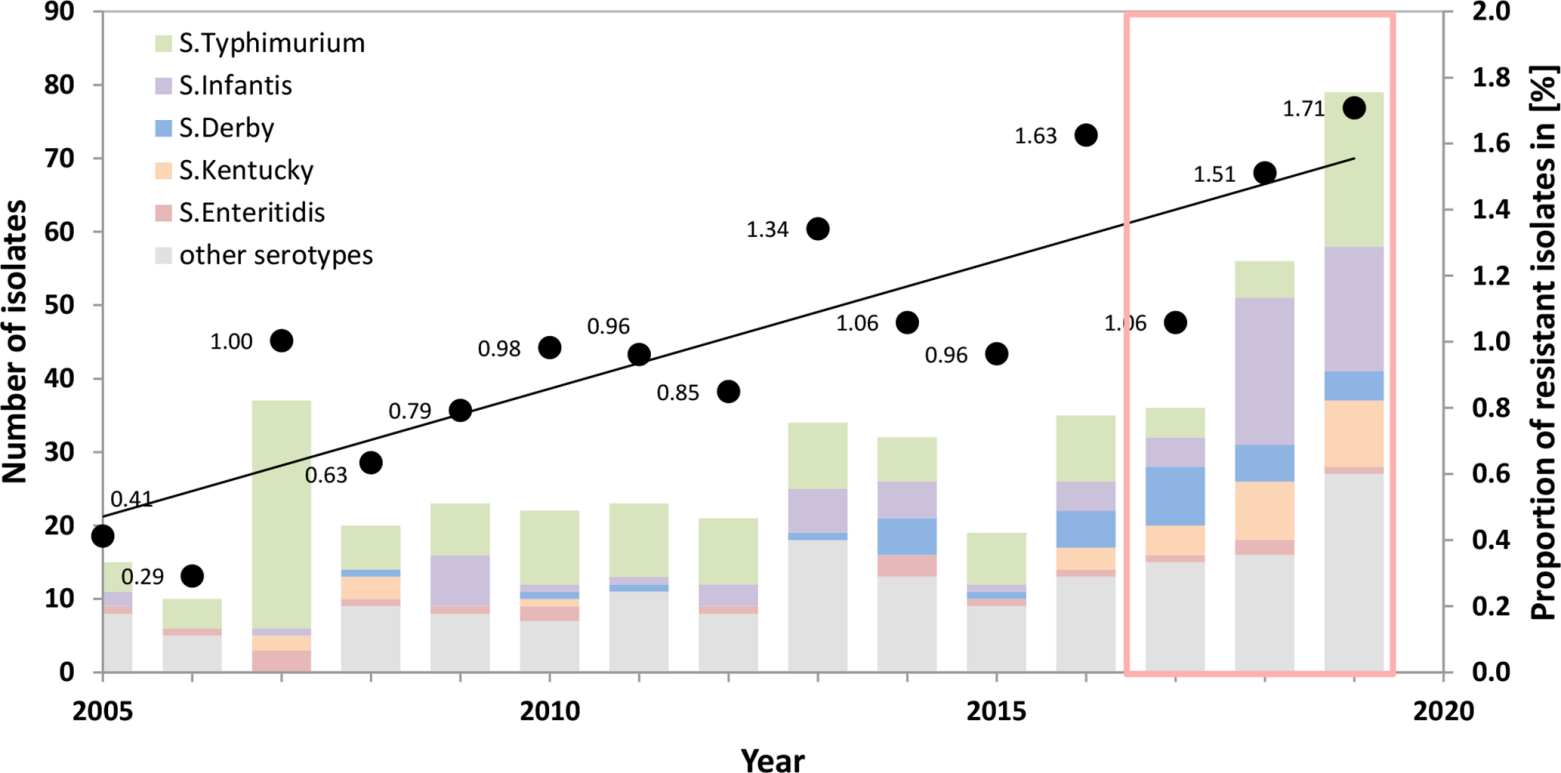
Increasing proportion of German clinical *S. enterica* isolates showing third generation cephalosporin resistance (cefotaxime and/or ceftazidime) from 2005 to 2019. The left axis indicates the absolute numbers of third generation cephalosporin-resistant isolates (coloured bars), the right axis indicates the proportion of third generation cephalosporin-resistant isolates in percent of all analysed isolates at the NRC (black dots). Isolates from 2017 to 2019 investigated in detail in this study are highlighted by the red box. The resistant *S*. Typhimurium isolates from 2019 included four representative isolates of an infection cluster.

In 2019, a proportion of 1.71 % of all analysed *S. enterica* were resistant to third generation cephalosporins, which was an increase compared to 1.06 % in 2017. To analyse whether the increasing trend is seen in a larger time frame, NRC data from the years 2005 to 2011 [11] and 2012 to 2016 were integrated into this analysis, using identical selection criteria. Indeed, the rising trend of third generation cephalosporin resistance was corroborated for the entire time frame from 2005 to 2019. The proportion of third generation cephalosporin resistance consistently rose from 0.41 % in 2005 to 1.71 % in 2019 (Fig. 1) (*P*=0.0002; correlation coefficient *r*=0.8318; Spearman rank correlation). In addition to resistance to third generation cephalosporins, 44 of the 168 isolates (26.2 %) also showed resistance to ciprofloxacin (Table S1) and 21 isolates (12.5 %) exhibited a combined resistance to ampicillin, chloramphenicol and trimethoprim/sulfamethoxazole (Table S1). No isolate was resistant to meropenem.

The 168 third generation cephalosporin resistant isolates were assigned to 34 different serovars, of which *S*. Infantis (*n*=41), *S.* Typhimurium (*n*=27), *S.* Kentucky (*n*=21), *S*. Derby (*n*=17) and *S*. Anatum (*n*=9) were most prevalent (Table S1 and S2).

### ESBL types CTX-M-1, CTX-M-14, and CTX-M-65 are the most abundant third generation cephalosporin resistance determinants

Whole genome sequence analyses including *in silico* detection of the resistance determinants revealed that for most of the isolates (*n*=163/166) the third generation cephalosporin resistance was mediated by ESBLs (*n*=147) or plasmid-borne AmpC β-lactamases (*n*=16) (Table S1). For three isolates no β-lactamase genes were detected and analyses of porin genes revealed wild-type variants without a sign of porin loss. Therefore, the underlying mechanism of resistance of these three isolates remained unclear (Table S1). The most prevalent ESBL genes in the isolate collection were *bla*_CTX-M-1_ (*n*=55), followed by *bla*_CTX-M-14_ (*n*=25, different variants) and *bla*_CTX-M-65_ (*n*=23) (Table 1). The most commonly detected AmpC β-lactamase gene was *bla*_CMY-2_ (*n*=13), only one isolate harboured *bla*_CMY-4_, one isolate *bla*_ACC-1_, and one isolate *bla*_DHA-1_. Further identified β-lactamase genes were: *bla*_OXA-1_ (*n*=1), *bla*_CARB-2_ (*n*=2) and 39 *bla*_TEM_ variants. Additionally, 38 isolates harboured plasmid-mediated quinolone resistance (PMQR) genes, mainly gene *qnrS1* (*n*=25). Interestingly, four isolates were positive for variants of the mobile colistin resistance genes *mcr-1.1* (*S.* Typhimurium*,* 17-02112)*, mcr-3.1* (*S.* Typhimurium, 19-03137) and *mcr-3.11* (*S.* Choleraesuis and *S.* Bovismorbificans, 17-00186 and 17-05354) (Table 1).

**Table 1.** ESBL and AmpC β-lactamase genes identified in the third generation cephalosporin resistant clinical *Salmonella* isolates from Germany, 2017 – 2019. Information on the co-occurrence of plasmid-mediated quinolone resistance (PMQR) genes and colistin resistance-mediating genes in the respective *Salmonella* isolates is given.

ESBL/AmpC gene	n	PMQR genes (n)	*Mcr* genes (n)	Majorly found in (n)
*bla*_CTX-M-1_*	55	*qnrS1* (4)		*S.* Infantis (15) *S.* Derby (17)
*bla*_CTX-M-2_	1			
*bla*_CTX-M-3_	3			
*bla*_CTX-M-9_	3			
*bla*_CTX-M-14_	25	*qnrS1* (3)	*mcr-1.1* (1), *mcr-3.11* (2)	*S.* Kentucky (18)
*bla*_CTX-M-15_	7	*qnrS1* (1)		
*bla*_CTX-M-32_	1			
*bla*_CTX-M-55_	14	*qnrS1* (6)	*mcr-3.1*(1)	
*bla*_CTX-M-65_	23			*S.* Infantis (22)
*bla*_TEM-52_	2	*qnrB19* (1)		
*bla*_SHV-12_	13	*qnrS1* (10), *qnrB2* (1), *qnrA1* (1)		*S.* Anatum (7)
*bla*_CMY-2_	13	*qnrS1* (1), *qnrB19* (4), *qnrB6* (1)		
*bla*_CMY-4_	1	*qnrA1* (1)		
*bla*_DHA-1_	1	*qnrB4* (1)		
*bla*_ACC-1_	1			

*Includes four representative isolates originating from the 2019 *S.* Typhimurium infection cluster.

**Fig. 2.**
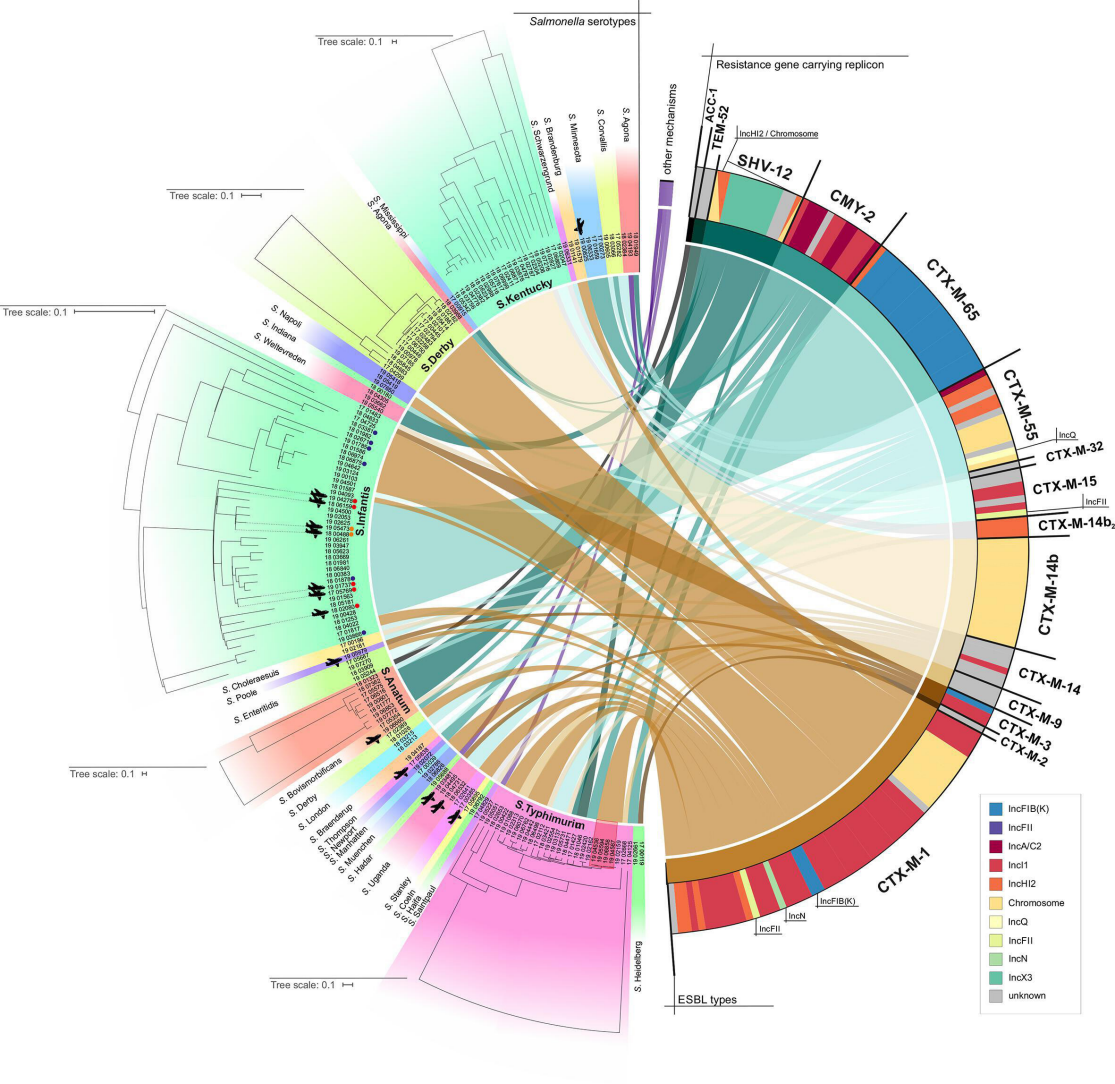
Attribution of the *Salmonella* serovar to the respective ESBL/AmpC gene of 166 third generation resistant clinical *Salmonella* isolates from Germany 2017 – 2019. The attributed resistance gene of each isolate is indicated via coloured links. The resistance gene carrying replicon is additionally indicated in the coloured bars next to the resistance gene (see legend). The respective phylogenetic relationships of *S*. Anatum, *S*. Derby, *S*. Infantis, *S*. Kentucky and *S*. Typhimurium are highlighted by independent phylogenetic trees. Four representative isolates belonging to the *S*. Typhimurium infection cluster are shown with a red background. Isolates with possible travel history are highlighted by airplanes and coloured dots, indicating the origin of the isolates (orange: South-East Asia, red: South America, blue: Germany). The figure was created with Circos [47].

Although a strict correlation of serovar and ESBL/AmpC type was not detected, some ESBL types were observed in high proportions in specific serovars. For example, *bla*_CTX-M-65_ (*n*=22/23) was mainly identified in *S.* Infantis. Additionally, *bla*_CTX-M-14_ was differentiated into three subgroups (*bla*_CTX-M-14_, *n*=6, *bla*_CTX-M-14b_, *n*=16 and *bla*_CTX-M-14b2_, *n*=3): While *bla*_CTX-M-14b_ was solely detected in *S.* Kentucky (Fig. 2, Table 1), the remaining *bla*_CTX-M-14_ variants were present in various serovars.

### Plasmids as the main carriers of genes encoding ESBLs and AmpC β-lactamases

For 137 of 163 of the ESBL/AmpC-positive isolates an attribution of the resistance gene to a respective genetic backbone / replicon was possible. The majority of the resistance genes was located on mobile genetic elements (*n*=105, 65.2 %), particularly on plasmids, irrespective of their hosting *S. enterica* serovar. However, a substantial number of isolates (*n*=32, 18.8 %) revealed chromosomal integration of the ESBL genes. The chromosomal integration of *bla*_CTX-M-14_ in all *S*. Kentucky isolates was identical as described by Coipan *et al*: *bla*_CTX-M-14_ was flanked by an upstream IS*Ecp1* element, downstream of the *hcp1* gene in the type VI secretion system region of the genome [14]. In all *S*. Derby isolates the integration of *bla*_CTX-M-1_ with an upstream IS*Ecp1,* as presumable mobilizing element, took place in a different genetic region than in *S.* Kentucky. *Bla*_CTX-M-1_ was integrated into the *kduI* gene, which was inferred from homology, causing a truncation of this pectin degradation pathway gene (accession no. A0A379QY07).

For 26 isolates it was only possible to recover the direct genetic environment but not to attribute the respective replicon type.

While for some of the ESBL genes a correlation of resistance gene and the gene carrying replicon was observed (*bla*_CTX-M-65_ and IncFIB(K) (22/23), *bla*_CTX-M-14b_ and chromosome (16/19), *bla*_CMY-2_ and IncI1 (6/13), *bla*_CMY-2_ and IncA/C (6/13)), other genes showed a more diverse genetic background. For example, *bla*_CTX-M-1_ was found on IncI1, IncHI2, IncN and IncQ plasmids.

### Predominance of emerging pESI plasmids in *S.* Infantis harbouring *bla*_CTX-M_

Detailed comparative analyses of *bla*_CTX-M-65_ carrying plasmids revealed a high sequence identity with the previously described pESI-like plasmid identified in *S.* Infantis. Specifically, 22 of 23 *bla*_CTX-M-65_ positive isolates showed high resemblance with the chimeric backbone of the pESI-plasmid p119944 (accession no. NZ_CP047882) and all belonged to the serovar *S*. Infantis. The non-matching isolate belonged to *S*. Indiana (18-00180) and the respective plasmid revealed no similarities to the pESI plasmid backbone. The integration of *bla*_CTX-M-65_ usually takes place upstream of a previously described drug and heavy metal resistance gene carrying region and this was also observed for the *S.* Infantis isolates in this study [26]. Further analyses showed that the presence of *bla*_CTX-M-1_ on a pESI-like plasmid backbone was observed only in two out of 15 *S*. Infantis isolates (17-01817 and 18-04022). We found that the integration of *bla*_CTX-M-1_ took place in the conserved IncI1 backbone structure of pESI. The remaining 13 *S*. Infantis isolates however exhibited a high sequence identity (>95 %) with the IncI1 plasmid p15078279 (accession no. MK181566), which was previously identified in *E. coli* [27]. Additionally, in one isolate (18-04833) *bla*_CTX-M-3_ was suspected to be located on a pESI-like plasmid. While confirmation of the insertion of *bla*_CTX-M-3_ into the pESI based on short read sequencing data was not possible, the sequence data however suggested the presence of the full pESI-like backbone. Additional mating experiments and subsequent S1-nuclease restriction and pulsed-field gel electrophoresis revealed transferability of the *bla*_CTX-M-3_ gene and its presence on an approx. 290 kb sized plasmid for the *bla*_CTX-M-3_ positive transconjugant of 18-04833. In comparison, the size of the transferred *bla* gene carrying plasmids of *bla* positive transconjugants of other pESI-like plasmid carrying strains, such as *S.* Infantis 17-01817 (*bla*_CTX-M-1_) and 19-02625 (*bla*_CTX-M-65_), was approx. 290 kb and approx. 315 kb, respectively.

### *bla*_CMY-2_ harbouring plasmids share high sequence similarity with IncI1 and IncA/C plasmids from Europe

AmpC β-lactamase CMY-2 was the main determinant of resistance to third generation cephalosporins present in 13 isolates (mainly serovars *S*. Minnesota *n*=4 and *S*. Typhimurium *n*=3). The *bla*_CMY-2_ gene was found to be located on plasmids of the replicon type IncI1 (*n*=6) and IncA/C replicon type (*n*=6) and for one isolate (19-00825) was not assignable. The plasmids of IncI1 replicon type showed a high sequence identity to two previously described variants: IncI1 belonging to the plasmid sequence type pST12 (accession no. CP012929) (*n*=4) and IncI1 belonging to pST2 (accession no. KT186369) (*n*=2) and shared >98 %, respective >99 % sequence identity with these reference plasmids. Isolates 17-05838 and 19-02072 resembled the backbone of IncA/C_2_ plasmids of type 1 (accession no. CP015835) [28], while the other four isolates (17-00273, 17-01659, 19-02661, 19-06333) showed a high similarity with a IncA/C reference plasmid (accession no. CP060509), which was identified in NTS isolates from Russia.

### The *S.* Typhimurium infection cluster isolates from 2019 harboured a novel plasmid carrying *bla*_CTX-M-1_

**Fig. 3.**
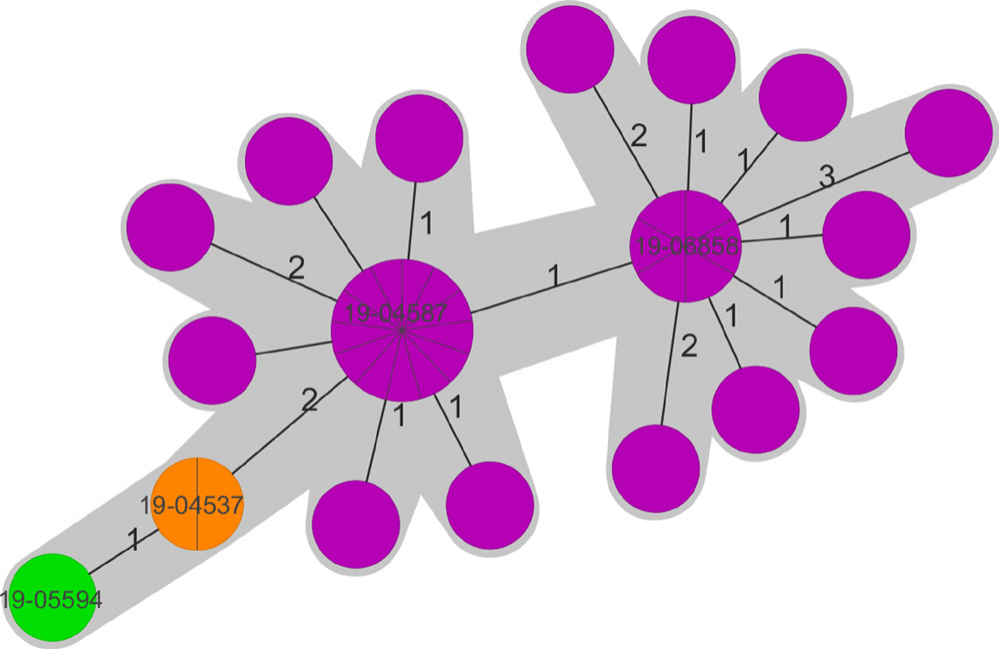
cgMLST-based Minimum Spanning Tree of 36 *S*. Typhimurium isolates of the 2019 infection cluster. Isolates with an identical IncHI2 plasmid structure are shown in purple, IncHI2 plasmid variants with the additional IS element in orange and the isolate with the unique IncI1 plasmid in green. Only the four representative isolates are highlighted with strain numbers.

As depicted above, a substantial part of the *S.* Typhimurium isolates (*n*=100) of the year 2019 originated from a single infection cluster in a regional setting. Genome analyses of 36 of these isolates were performed and confirmed their close genetic relationship. The maximum distance in the cgMLST analysis were five alleles; the majority of the genomes depicted a distance of zero or one alleles (Fig. 3). The resistance gene content and plasmid replicon analyses showed almost identical patterns (*bla*_CTX-M-1_ : 36/36, *qnrS1* : 35/36, IncHI2 replicon type: 35/36). The identified resistance gene carrying plasmid showed no sequence similarity to previously published *bla* gene carrying plasmids. Interestingly, one isolate (19-05594) showed absence of the *qnrS1* gene and revealed a unique plasmid replicon background (IncI1), indicating a potential independent plasmid acquisition event. Two further isolates (19-04536 and 19-04537) exhibited a slight alteration of the *bla*_CTX-M-1_ carrying plasmid, varying in IS element integration sites. One representative isolate from each group (19-04587, 19-06858, 19-04536: IncHI2 plasmid alteration; 19-05594: IncI1 plasmid) was included in all here presented analyses (Figs. 2 and 3).

### No strict correlation of ESBL genes with certain serovars was observed, but some serovar/resistance gene combinations, such as *S*. Infantis with *bla*_CTX-M-1_ or *bla*_CTX-M-65_ or *S*. Kentucky with *bla*_CTX-M-14b_, were detected most frequently

cgMLST-based analysis revealed that the majority of the isolates showed no close phylogenetic relationship but clustered according to the serovar. However, among the serovars various (distinctly related) subclades were observed (Fig. 2, subclades shown for *S*. Anatum, *S*. Derby, *S*. Infantis, *S*. Kentucky, and *S*. Typhimurium). As expected, the four representative *S*. Typhimurium isolates from the infection cluster formed one cluster (sequence type ST19) (Figs 2 and 3), while the other *S*. Typhimurium isolates belonged to the ST34. Linking data on *bla* gene and plasmid content to phylogenetic information, a tendency regarding the presence of specific ESBL genes in certain serovars was observed. The most apparent correlations were seen for *S*. Infantis with *bla*_CTX-M-1_ and *bla*_CTX-M-65_, *S*. Typhimurium with *bla*_CTX-M-1_, *S*. Derby with *bla*_CTX-M-1_ and *S*. Kentucky with *bla*_CTX-M-14b_ (Fig. 2).

In depth cgMLST analyses of the *S*. Infantis clade showed that these strains can be differentiated into at least two different subclades: subclade one exhibited the IncI1 *bla*_CTX-M-1_ carrying plasmid while subclade two is associated with *bla*_CTX-M-65_ and *bla*_CTX-M-1_ carrying pESI-like plasmids (Fig. S1). Additionally, one isolate (18-04833) exhibited *bla*_CTX-M-3_ presumably integrated into the pESI backbone. Little information on travel background of patients was available. However, for thirteen of the 41 *S.* Infantis isolates a definite exposition region was determined. Interestingly, especially isolates from patients from the subclade carrying the *bla*_CTX-M-65_ pESI-like plasmid exhibited travel history to South East Asia or South America (Figs 2 and S1).

## Discussion

The analyses of clinical NTS from Germany showed a trend towards increasing resistance proportions for third generation cephalosporins (Fig. 1). This observation is in accordance with the worldwide emergence of antibiotic resistance in NTS. European data for clinical NTS isolates (period 2018 – 2019) by EFSA and ECDC [5] uncovered high resistance rates for different classes of antibiotics, especially ampicillin, sulfonamides, and tetracyclines. Increasing resistance was further reported for fluoroquinolones, while resistance to third generation cephalosporins was still on low levels but with rising proportions in certain serovars [5]. In Germany, five serovars (*S*. Typhimurium, *S*. Derby, *S*. Kentucky, *S.* Infantis and *S*. Anatum) accounted for over 65 % of the resistant isolates in this study [5, 29, 30]. *S.* Enteritidis, while being one of the most prevalent *S. enterica* serovars, remains mostly susceptible to third generation cephalosporins [31].

In the present study we identified ESBL and AmpC β-lactamase genes as most important determinants of third generation resistance in clinical NTS. These observations are in concordance to reports from the EU, where in 2018/19 ESBLs were reported in 16 different serovars (among those *S*. Infantis, *S*. Kentucky, *S*. Typhimurium) and AmpC β-lactamases in ten different serovars, mostly in *S*. Anatum, *S.* Bredeny and *S*. Thompson [5]. The majority of isolates in this study harboured ESBL genes encoding CTX-M enzymes (*n* = 132; 80.0%) with CTX-M-1, CTX-M-14 and CTX-M-65 as the most prevalent types. Similar results were found in studies on *S. enterica* and *E. coli* isolated from animals, especially poultry [11, 32, 33] and in reports from the EU, where, if tested by member states, the ESBL variants CTX-M-1, CTX-M-14/14b and CTX-M-9 were frequently reported [5]. In the present study *bla*_CTX-M-1_ was detected in various serovars, including *S*. Typhimurium, *S*. Infantis and *S*. Derby. However, *bla*_CTX-M-14b_ and *bla*_CTX-M-65_, were almost exclusively found in *S.* Kentucky and *S*. Infantis, respectively. Detailed sequence analyses of the 23 *bla*_CTX-M-65_ carrying plasmids of the study isolates revealed a high sequence identity among them: out of the 23 isolates, 22 belonged to the serovar *S*. Infantis and showed an identical plasmid structure, which additionally was highly similar to the chimeric backbone of the pESI-plasmid p119944 (accession no. NZ_CP047882).

In recent studies the spread of third generation cephalosporin resistant *S*. Infantis in food-producing animals associated with the presence of a specific resistance plasmid type, the pESI-like megaplasmid, was reported [15, 34, 35]. This plasmid type/backbone was associated with the carriage of ESBL genes (*bla*_CTX-M-65_ and *bla*_CTX-M-1_), several other resistance genes (*tet(A), sul1, dfrA1, dfrA14, aadA1*) and heavy metal resistance genes [26]. The pESI-like megaplasmid was first identified by Aviv *et al.* in Israel[34], however, not yet harbouring third generation cephalosporin resistance genes. Subsequently findings of pESI harbouring third generation cephalosporin resistance genes were reported from Italy [15], the U.S. [28], Denmark, Netherlands and the UK [15, 34–36]. In 2018, Brown *et al*. observed the linkage of *S*. Infantis pESI-like plasmids carrying *bla*_CTX-M-65_ in the USA with travel activity to Latin America [37]. Despite too little information on the travel history of the patients in our study for statistical analyses, the present data partly indicate a correlation between travel activity (Asia and South America) and *bla*_CTX-M-65_ harbouring *S*. Infantis.

While the pESI plasmid with *bla*_CTX-M-65_ was highly prevalent in *S*. Infantis strains from Germany, only two of 15 *S*. Infantis strains exhibiting *bla*_CTX-M-1_ (17-01817 and 18-04022) harboured the resistance gene on the pESI plasmid. In Germany the occurrence of pESI was described in *S*. Infantis isolates from broiler farms since the 2010s [38]. However, these plasmids did not carry ESBL genes and were identified in *S*. Infantis strains of another lineage (ST2283) than the *bla*_CTX-M-1_ carrying isolates from this study (ST32). In our study one additional isolate of *S.* Infantis exhibited *bla*_CTX-M-3_ on a pESI-like plasmid, highlighting the potential of this particular plasmid backbone to acquire further resistance genes. Phylogenetic analysis revealed that isolates harbouring pESI with *bla*_CTX-M-3_, *bla*_CTX-M-65_ and *bla*_CTX-M-1_ are more related among each other than to the clade of the non-pESI *bla*_CTX-M-1_ carrying *S*. Infantis isolates. It can be speculated, that certain genetic factors enable the plasmid to establish in this specific *S.* Infantis *bla*_CTX-M-1/-65_ clade. The presence of multiple post-segregational killing determinants on the plasmid may be of advantage for this process [34].

The majority of *S*. Infantis isolates with *bla*_CTX-M-1_ (13/15) exhibited a highly similar plasmid background to an IncI1 plasmid backbone. Identical or highly similar *bla*_CTX-M-1_ carrying plasmids were identified in the present study also in *S*. Derby, *S*. Enteritidis, *S.* Typhimurium, *S*. Stanley and *S*. Brandenburg. In contrast to the pESI plasmid, which appears to be more adapted to the *S.* Infantis host - but still can be transferred into other *S. enterica* serovars and *E. coli* [15, 39] - these non-pESI IncI1 plasmids exhibited a wider host range and were also reported in previous studies in *E. coli* [27].

Further resistance genes detected in this study were *bla*_CTX-M-15_ and *bla*_CMY-2_. ESBL type CTX-M-15, which is highly prevalent in *E. coli* from outpatient and hospitalized patients in Germany [40]*,* was identified only in seven clinical *S. enterica* isolates of different serovars. AmpC β-lactamase CMY-2, which was detected in <1 % of clinical *E. coli* isolates with third generation cephalosporin resistance in Germany [41] were detected in 12 *S. enterica* isolates in the present study (7.45 %). The observed higher proportions of CTX-M-1 and CMY-2 genes with comparable lower proportions of CTX-M-15 in our clinical *S. enterica* isolates may on the one hand indicate a possible transmission route between livestock and humans via the food chain. The active acquisition of plasmids via horizontal gene transfer along the food production chain seems rational, as identical resistance gene carrying plasmids were observed in *S. enterica* of different serovars. This is strengthened by the observation of identical resistance gene carrying plasmids in *E. coli, Klebsiella pneumoniae* and other Enterobacterales species in Germany, Europe, and worldwide [42–44]. Furthermore, it has been reported that approx. 6 % of the healthy population of Germany carries ESBL positive *E. coli* [45]. Ingestion of other ESBL-positive Enterobacterales and transfer of resistance genes from commensal bacteria therefore might have an impact on the treatment options of severe salmonellosis, as the resistance genes may be acquired during treatment [46]. However, since CTX-M-15 is the most common ESBL type in *E. coli*/* K. pneumoniae* in the human population, these events might be limited in occurrence rather than being the main route for ESBL gene transmission.

We conclude that third generation cephalosporin resistance, is on the rise among clinical *S. enterica* isolates in Germany and that its occurrence in various *S. enterica* serovars is likely due to multiple acquisition events of plasmids. However, specific adaption processes, as observed for *S*. Infantis with pESI plasmids harbouring different *bla*_CTX-M_ genes, could facilitate the spread of resistant *S. enterica* isolates.

## Supplementary Data

Supplementary material 1

Supplementary material 2
