# Supplementary material for: Third generation cephalosporin resistance in clinical non-typhoidal Salmonella enterica in Germany and emergence of bla CTX-M-harbouring pESI plasmids
Source: Microb Genom. 2021 Oct 25;7(10):000698. doi: 10.1099/mgen.0.000698 (PMC8627203; doi:10.1099/mgen.0.000698)
Supplement: Supplementary material 1 [file mgen-7-0698-s001.pdf]

Supplementary Material for

3rd generation cephalosporin resistance in clinical non-typhoidal *Salmonella enterica* in Germany and emergence of *bla*$_{CTX-M}$-harboring pESI plasmids

Michael Pietsch, Sandra Simon, Anika Meinen, Eva Trost, Sangeeta Banerji, Yvonne Pfeifer, Antje Flieger

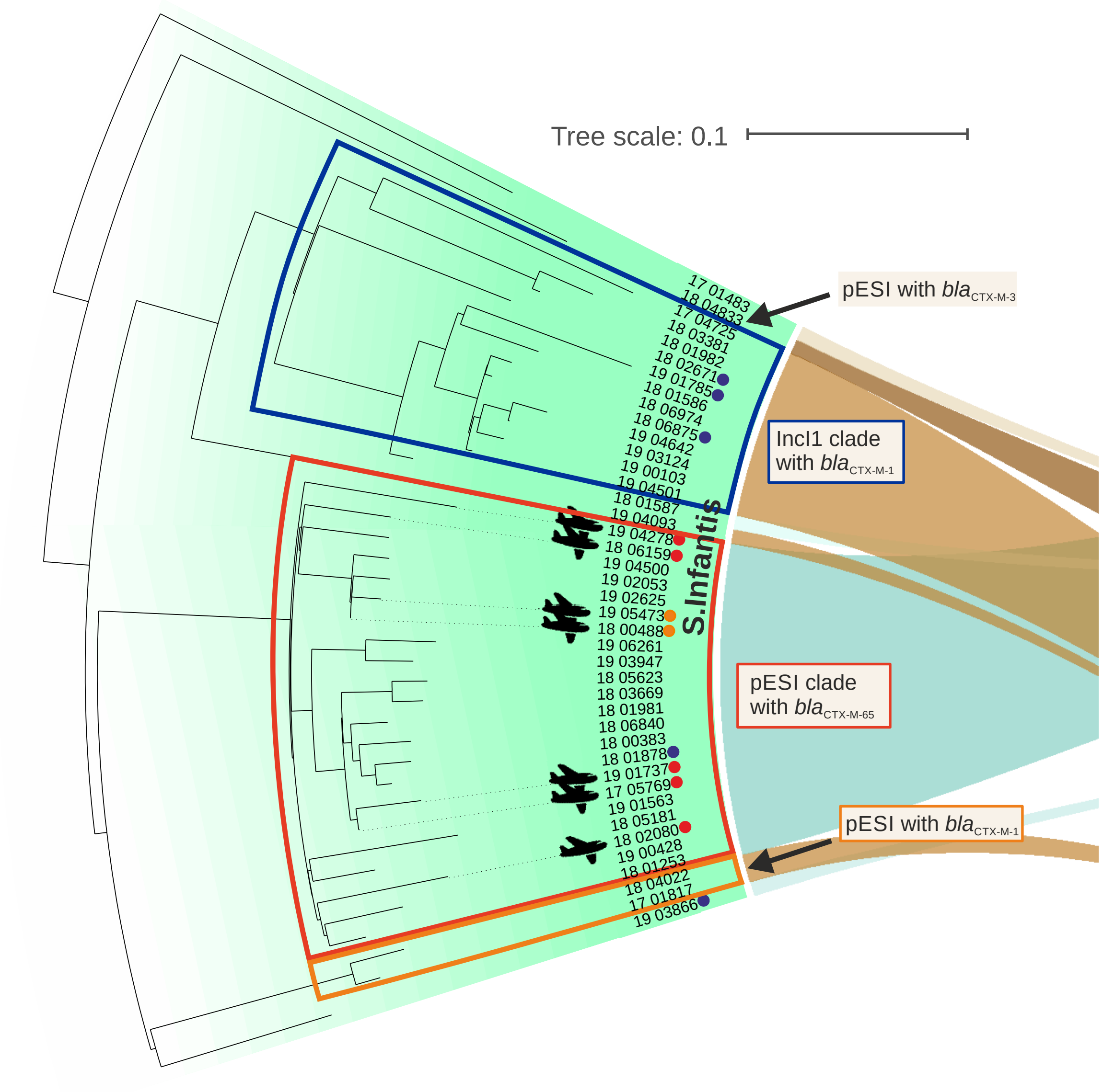

Figure S1: *Separation of S. Infantis into specific subclades, associating with different resistance genes and plasmid types. Isolates with possible travel history are highlighted by airplanes and colored dots, indicating the origin of the isolates (orange dots: South-East Asia, red dots: South America, blue dots: Germany)*

Table S2: *Number of cephalosporin-resistant clinical Salmonella isolates analyzed in the years 2012 - 2019 at the German National Reference Centre for Salmonella and other Enteric Bacterial Pathogens (NRC). The proportion respective to all analyzed isolates for cephalosporin-resistant Salmonella of all serovars, and specifically for S. Infantis, S. Typhimurium, S. Kentucky and S. Derby are given. Isolates showing phenotypic resistance to cefotaxime and/or ceftazidime (CAZ) according to EUCAST criteria are considered.*

	**Analyzed *Salmonella* of all serovars**			***S.* Infantis**		***S.* Typhimurium**		***S.* Kentucky**		***S.* Derby**		**remaining serovars**	
**Year**	***Isolates***	***CAZ resistant***	***%***	***n* *CAZ res.***	**Proportion [%] *n* of all isolates**	***n* *CAZ res.***	**Proportion [%] *n* of all isolates**	***n* *CAZ res.***	**Proportion [%] *n* of all isolates**	***n* *CAZ res.***	**Proportion [%] *n* of all isolates**	***n* *CAZ res.***	**Proportion [%] *n* of all isolates**
2012	2,471	21	0.85	3	4.0 n=73	9	1.0 (n=948)	0	0 (n=29)	0	0 (n=75)	9	0,7 (n=1,346)
2013	2,532	34	1.34	6	1.5 n=395	9	0.9 (n=1,034)	0	0 (n=12)	1	2.1 (n=48)	18	1.7 (n=1,043)
2014	3,023	32	1.06	5	4.2 n=120	7	0.9 (n=778)	0	0 (n=13)	5	2.5 (n=199)	15	0.8 (n=1,913)
2015	1,971	19	0.96	1	0.8 n=123	7	1.2 (n=595)	3	15.8 (n=19)	1	3 (n=33)	7	0.6 (n=1,201)
2016	2,153	35	1.63	4	2.4 n=164	9	1.3 (n=652)	4	10 (n=40)	5	8.1 (n=62)	13	1 (n=1,235)
2017	3,399	36	1.06	4	2.5 n=159	4	0.5 (n=922)	4	10.3 (n=39)	8	15.1 (n=53)	16	0.7 (n=2,226)
2018	3,706	56	1.51	20	8 n=250	5	0.6 (n=881)	8	9.4 (n=85)	5	3.5 (n=143)	18	0.8 (n=2,347)
2019	4,625	79	1.71	17	5.2 n=326	21	1.6 (n=1,235)	9	13.4 (n=67)	4	3.3 (n=123)	28	1 (n=2,874)
